# High Pulsatile Load Decreases Arterial Stiffness: An *ex vivo* Study

**DOI:** 10.3389/fphys.2021.741346

**Published:** 2021-10-22

**Authors:** Cédric H. G. Neutel, Giulia Corradin, Pauline Puylaert, Guido R. Y. De Meyer, Wim Martinet, Pieter-Jan Guns

**Affiliations:** ^1^Laboratory of Physiopharmacology, Faculty of Medicine and Health Sciences, University of Antwerp, Campus Drie Eiken, Antwerp, Belgium; ^2^Department of Pharmaceutical and Pharmacological Sciences, University of Padua, Padua, Italy; ^3^Laboratory of Physiopharmacology, Faculty of Pharmaceutical, Biomedical and Veterinary Sciences, University of Antwerp, Campus Drie Eiken, Antwerp, Belgium

**Keywords:** pulse pressure, arterial stiffness, biomechanics, infrarenal aorta, thoracic descending aorta, VSMC

## Abstract

Measuring arterial stiffness has recently gained a lot of interest because it is a strong predictor for cardiovascular events and all-cause mortality. However, assessing blood vessel stiffness is not easy and the *in vivo* measurements currently used provide only limited information. *Ex vivo* experiments allow for a more thorough investigation of (altered) arterial biomechanical properties. Such experiments can be performed either statically or dynamically, where the latter better corresponds to physiological conditions. In a dynamic setup, arterial segments oscillate between two predefined forces, mimicking the diastolic and systolic pressures from an *in vivo* setting. Consequently, these oscillations result in a pulsatile load (i.e., the pulse pressure). The importance of pulse pressure on the *ex vivo* measurement of arterial stiffness is not completely understood. Here, we demonstrate that pulsatile load modulates the overall stiffness of the aortic tissue in an *ex vivo* setup. More specifically, increasing pulsatile load softens the aortic tissue. Moreover, vascular smooth muscle cell (VSMC) function was affected by pulse pressure. VSMC contraction and basal tonus showed a dependence on the amplitude of the applied pulse pressure. In addition, two distinct regions of the aorta, namely the thoracic descending aorta (TDA) and the abdominal infrarenal aorta (AIA), responded differently to changes in pulse pressure. Our data indicate that pulse pressure alters *ex vivo* measurements of arterial stiffness and should be considered as an important variable in future experiments. More research should be conducted in order to determine which biomechanical properties are affected due to changes in pulse pressure. The elucidation of the underlying pulse pressure-sensitive properties would improve our understanding of blood vessel biomechanics and could potentially yield new therapeutic insights.

## Introduction

Large artery stiffness is a strong predictor of cardiovascular events and all-cause mortality and directly impacts blood pressure. Evidently, measurement of arterial stiffness *in vivo* and *ex vivo* has gained a lot of interest (Vlachopoulos et al., 2010; Leloup et al., 2016; Avolio et al., 2018). Determining pulse wave velocity (PWV) is the cornerstone for the *in vivo* measurement of arterial stiffness (Butlin et al., 2020). However, PWV does not make a distinction between the contribution of different components of the vessel wall to the overall stiffness. *Ex vivo* measurements of arterial stiffness may provide more insight into the mechanisms that cause blood vessel stiffening. Such *ex vivo* measurements can reveal important (altered) biomechanical properties of blood vessels, which can advance the research field of “vascular mechanomedicine” (Naruse, 2018).

Measuring blood vessel stiffness *ex vivo* can either be done in a static or a dynamic manner. However, the biomechanical behavior of blood vessels differs between stationary and oscillatory experiments (Cox, 1974; Glaser et al., 1995; Lénárd et al., 2000). Therefore, assessing arterial stiffness under pulsatile conditions is favorable, as it is more representative of physiological conditions. Pulsatile *ex vivo* setups such as the bi-axial stretching setup of van der Bruggen M. M. et al. (2021) or the dynamic intraluminal pressure testing system by Santelices et al. (2007) are able to produce quasi-physiological pressure waveforms. In these setups, pulsatile load/stretch can be mimicked in *ex vivo* conditions, allowing a dynamic assessment of blood vessel stiffness.

Our research group previously developed a wire myography-based organ bath setup, called ROTSAC, that enables imposing different pulsatile stretching conditions (Leloup et al., 2016). Using this setup, the role of vascular smooth muscle cells (VSMCs) and endothelial cells (ECs) in arterial viscoelasticity was extensively studied (Leloup et al., 2017, 2019, 2020; De Munck et al., 2020; Bosman et al., 2021). In these experiments, the pulsatile load was kept at a pulse pressure of 40 mmHg to mimic the physiological condition. However, the amplitude of the pulsatile stretch is known to modulate mechanosensitive properties of the blood vessel, such as contractile properties, stress fiber alignment, cell morphology and gene expression in both ECs and VSMCs (Birukov, 2009; Haghighipour et al., 2010; Hsu et al., 2010). Moreover, the viscoelastic properties of the arterial wall, which modulate the stiffness of the vessel, can be affected by altered dynamic loading conditions (Tan et al., 2016; Xiao et al., 2017; Butlin et al., 2020). Therefore, instead of assessing the “pressure”–“strain” relationship at a single fixed pulse pressure, altering the stretch amplitude/pulse pressure could reveal other biomechanical responses which would otherwise be neglected.

In the present study, we investigated how altering the amplitude of the pulsatile stretch (i.e., the pulsatile load) *ex vivo* affects the measurement of arterial stiffness. We aimed to illustrate how VSMC function and arterial viscoelastic properties are affected with altered loading conditions. Specifically, VSMC contraction and relaxation were studied under different pulse pressures to assess whether their functionality is dependent on the pulsatile load. Finally, we compared the descending aorta with the infrarenal aorta in this model to assess differential effects of pulsatile stretch on an elastic and muscular artery, respectively.

## Materials and Methods

### Mice and Tissue Preparation

Seven male C57BL/6J mice (6 months old; Charles River Laboratories, France) were used in the present study. All animals were housed in the animal facility of the University of Antwerp in standard cages with 12 h-12 h light-dark cycles and had free access to regular chow and tap water. The animals were euthanized by perforating the diaphragm while under anesthesia [sodium pentobarbital (Sanofi, Belgium), 75 mg/kg i.p.]. The descending and infrarenal aorta were carefully removed and stripped of adherent tissue. The tissue was cut into segments of 2 mm. In all experiments, segments from the same anatomical location were used in order to minimize variability due to the heterogeneity in blood vessel composition along the aortic tree. The segments were immersed in Krebs Ringer (KR) solution (37°C, 95% O_2_/5% CO_2_, pH 7.4) containing (in mM): NaCl 118, KCl 4.7, CaCl_2_ 2.5, KH_2_PO_4_ 1.2, MgSO_4_ 1.2, NaHCO_3_ 25, CaEDTA 0.025 and glucose 11.1. The study was waived by the local ethics committee, according to article 3 of the EU legislation (L 276/38, 2010).

### Rodent Oscillatory Tension Set-Up to Study Arterial Compliance (ROTSAC)

The *ex vivo* stiffness of the aortic vessels was determined *via* ROTSAC measurements as previously described (Leloup et al., 2016). In brief, aortic segments were mounted between two parallel wire hooks in 10 mL organ baths. Force and displacement of the upper hook were controlled and measured with a force-length transducer. The segments were continuously stretched between alternating preloads, corresponding to a “diastolic” and “systolic” transmural pressure at a frequency of 10 Hz. The Laplace relationship was used to calculate the transmural pressure. At any given pressure, calibration of the upper hook allowed the calculation of the vessel diameter (both systolic and diastolic diameter) and the Peterson pressure-strain modulus of elasticity (Ep). The Ep was calculated as follows:


$$E\,p=D_{0}.\frac{△\,P}{△\,D}$$

**FIGURE 1:**
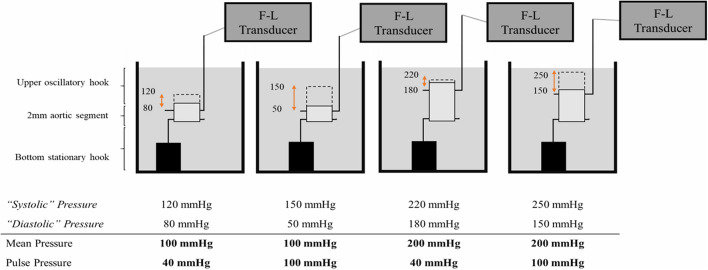
Altering pulsatile load *ex vivo*. To increase the pulse pressure at equal mean pressure, the diastolic and systolic pressures are decreased and increased, respectively, with equal increments. A segment that oscillates between 80 mmHg diastolic pressure and 120 mmHg systolic pressure, is exposed to a mean pressure of 100 mmHg and a pulse pressure of 40 mmHg. Alternatively, a segment that oscillates between 50 mmHg diastolic pressure and 150 mmHg systolic pressure, is exposed to an equal mean pressure of 100 mmHg but an increased pulse pressure of 100 mmHg. F-L Transducer = Force-Length Transducer.

With D_0_ the diastolic diameter. To dissect how pulse pressure affects the stiffness of the blood vessel, the amplitude of the oscillations was changed to generate higher or lower pulsatile stretches. However, mean pressure is an important regulator of *ex vivo* stiffness (Leloup et al., 2016). Therefore, altering pulse pressure should be done in such a manner that the mean pressures between pulse pressures are equal (Figure 1). In our setup, the duration of the diastolic and systolic phase are equal (50 ms for each phase). Therefore, mean transmural pressure is calculated as:


$$M\,e\,a\,n\,P\,r\,e\,s\,s\,u\,r\,e=\frac{\left(D\,i\,a\,s\,t\,o\,l\,i\,c\,P\,r\,e\,s\,s\,u\,r\,e+S\,y\,s\,t\,o\,l\,i\,c\,p\,r\,e\,s\,s\,u\,r\,e\right)}{2}$$

To increase pulse pressure, diastolic pressures were decreased and systolic pressures were increased in equal increments, leading to different pulse pressures, but equal mean pressures. Hence, the effect of a larger or smaller pulse can be assessed in isobaric conditions. The mean pressures were incrementally increased for all pulse pressures to assess the relationship between the non-linear increase in pressure-mediated stiffness and pulse pressure. All the measurements were conducted on the same aortic segment in order to make paired comparisons. A KCl solution (50 mM in Krebs) as well as the NO donor diethylamine NONOate (DEANO, 2 μM) were used to elicit VSMC contraction and relaxation, respectively. The resulting stiffness was measured at steady state conditions (i.e., 20 min after contraction or relaxation). The segments were washed three times after VSMC contraction in order to remove KCl before administering DEANO.

### Experimental Pulse Pressure Protocol

Five different pulse pressures were chosen to be implemented in the experimental protocol. The pulse pressure of 40 mmHg was considered as “physiological” pulse pressure and was therefore used as a reference for the other pulse pressures. One sub-physiological and three supra-physiological pulse pressures were included in the protocol (20 mmHg and 60, 80, and 100 mmHg, respectively). In this protocol, the mean pressure was increased from 80 to 200 mmHg in steps of 20 mmHg (see Supplementary Figure 1). The same pulse pressure protocol was applied to each vessel segment in three different conditions (Krebs, KCl, and DEANO). There was a waiting period of 30 min between conditions. Unless otherwise mentioned, segments from the thoracic descending aorta (TDA) were used in this study.

### Statistics

All results are expressed as mean ± SEM with n representing the number of mice. Statistical analyses were performed in GraphPad Prism 7.0. Statistical tests are mentioned in the figure and/or table legends. Significance was accepted at *P* < 0.05.

## Results

### Pulse Pressure Modulates the Non-linear Pressure-Stiffness Relationship

**FIGURE 2:**
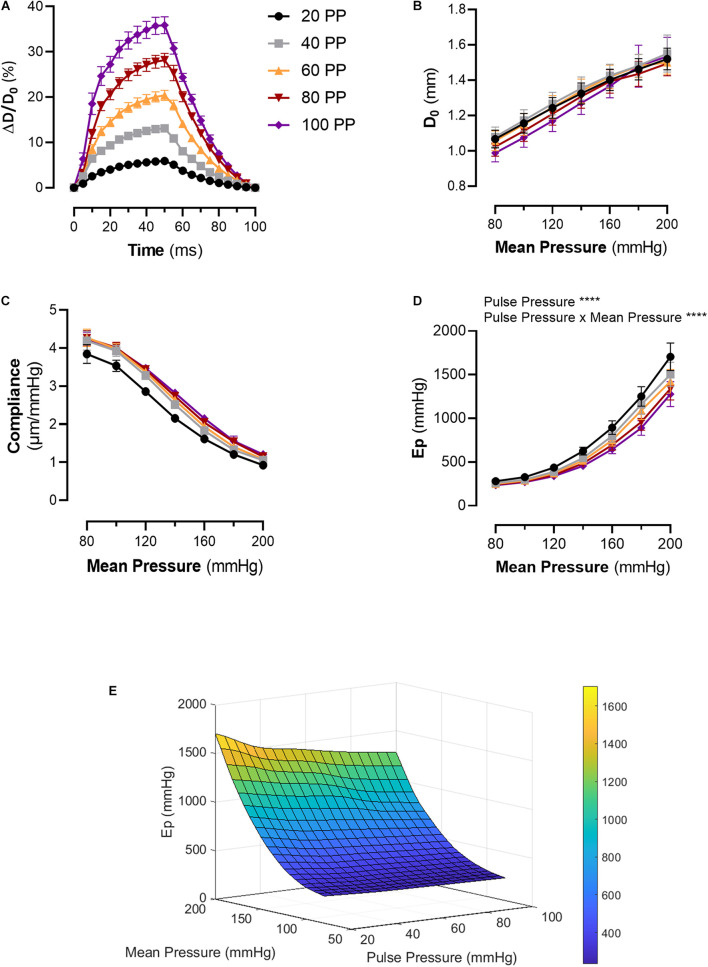
Pulsatile load modulates the pressure-stiffness relationship. **(A)** Mean distension of a single cycle (100 ms) of thoracic aorta segments at different pulse pressures. Tracings were downsampled to 5 ms intervals. **(B)** Diastolic diameter is altered by the applied pulse pressure, as expected due to the nature of the setup. **(C)** The relationship between compliance, pulse pressure and mean pressure. **(D)** The pressure-stiffness relationship is modulated by the pulse pressure. At low pulse pressures, the pressure-mediated increase in Ep is significantly augmented, while the inverse is true for increasing pulse pressures. **(E)** Surface plot representing the mean interpolated data. *n* = 7. Ep, Peterson’s modulus of elasticity; PP, pulse pressure.

Increasing pulse pressure augmented the maximum distension of the thoracic aortic segment (Figure 2A). The maximum distension (%) for pulse pressures of 20, 40, 60, 80, and 100 mmHg at a mean pressure of 100 mmHg were 6 ± 0.3, 13 ± 0.6, 20 ± 1, 28 ± 2, and 36 ± 2, respectively. Changing pulse pressure, alters both the D0 and the compliance (Figures 2B,C). Increasing mean pressure increases the Ep (Figure 2D). The pulse pressure significantly changed the pressure-stiffness relationship (Figures 2D,E and Table 1). Ep for each pulse pressure was different at every mean pressure (*p* < 0.0001), meaning that pulse pressure alters the stiffness of the same blood vessel (Figure 2D). Statistical comparisons in Table 1 were made to 40 mmHg pulse pressure, which was considered as the basal, physiological pulse pressure. Additionally, the interaction between pulse pressure and pressure was significant (*p* < 0.0001). A sub analysis was conducted for different pulse pressures at a mean pressure of 100 mmHg and demonstrated that pulse pressure significantly affects both D0 (*p* < 0.0001) and ΔP/ΔD (*p* = 0.001) (Supplementary Figure 2). This indicates that changes in Ep reflect both changes in diastolic diameter and altered compliance.

**TABLE 1:** Effect of differential pulse pressure on blood vessel stiffness.

**Mean pressure (mmHg)**	**Peterson’s modulus (Ep, mmHg)**				
	**20 PP**	**40 PP**	**60 PP**	**80 PP**	**100 PP**
80	(280 ± 9)**	(259 ± 8)	(248 ± 7)**	(241 ± 7)**	(234 ± 7)**
100	(328 ± 11)***	(298 ± 10)	(288 ± 12)**	(277 ± 11)***	(270 ± 10)***
120	(437 ± 23)***	(388 ± 22)	(371 ± 22)**	(353 ± 20)***	(338 ± 18)***
140	(625 ± 45)***	(545 ± 42)	(516 ± 41)***	(484 ± 36)***	(454 ± 31)***
160	(894 ± 79)**	(798 ± 71)	(748 ± 65)***	(695 ± 60)***	(645 ± 50)**
180	(1250 ± 112)**	(1158 ± 111)	(1089 ± 105)**	(958 ± 96)**	(890 ± 84)**
200	(1705 ± 158)*	(1502 ± 140)	(1424 ± 136)*	(1339 ± 130)**	(1277 ± 141)**

Data is expressed as (Mean ± SEM), *n* = 7. Repeated measures two-way ANOVA with Sidak *post hoc* test for multiple comparisons (comparisons are made with 40 PP as reference). *p < 0.05; **p < 0.01; ***p < 0.001. PP, pulse pressure (mmHg).

### Pulse Pressure Modulates Vascular Smooth Muscle Cell Reactivity

**FIGURE 3:**
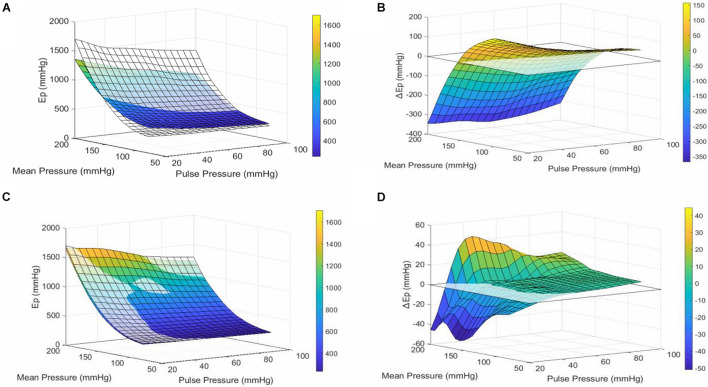
Pulse Pressure alters VSMC reactivity. **(A)** Surface plot of the mean interpolated raw data. The transparent surface plot is the uncontracted, basal state of the aortic segment, while the colored surface plot shows the same segment in 50 mM KCl-induced contracted state. At low mean pressures (80–140 mmHg), contraction of VSMCs makes the segment stiffer, while at high mean pressures (140–200 mmHg), the vessel is less stiff when contracted. This effect is consistent for all pulse pressures. **(B)** Surface plot of the mean interpolated data of the difference in stiffness between contracted and uncontracted state (contracted–basal). **(C)** Surface plot of the mean interpolated raw data. The transparent surface plot is the uncontracted, basal state of the aortic segment while the colored surface plot shows the same segment treated with 2 μM DEANO. **(D)** Surface plot of the mean interpolated data of the difference in stiffness (Ep) between relaxed and basal state (relaxed–basal). *n* = 7. Ep, Peterson’s modulus of elasticity; ΔEP, EP_contraction_–EP_uncontracted_; PP, pulse pressure.

The Pulse pressure-pressure-stiffness relationships were also investigated following constriction of aortic segments by depolarization with high extracellular K + or following removal of basal tonus with exogenous NO (DEANO) (Figure 3). The results for contraction and relaxation are shown in Tables 2, 3, respectively. We previously reported that VSMC contraction increases stiffness at low, physiological pressures, but decreases stiffness at higher, non-physiological pressures (Leloup et al., 2019). The comparisons between 50 mM KCl (50K) contraction induced stiffness at different pulse pressures and their uncontracted state are listed in Table 2. At pulse pressures of 20 and 40 mmHg, 50K increased the stiffness significantly at “low” mean pressures (80–140 mmHg) and decreased stiffness significantly at “high” mean pressures (180–200 mmHg) (Figure 3A). For the pulse pressures 60, 80, and 100 mmHg, 50K only decreased the stiffness significantly (*p* < 0.0001) at “high” mean pressures (160–200 mmHg). After 50K elicited contraction, all measurements (Ep) were significantly (*p* < 0.0001) different from each other. The ΔEp was significantly different between 20 and 40 mmHg pulse pressure, at the “low” mean pressures, indicating a stronger response to 50K in the former (Figure 3B).

**TABLE 2:** Blood vessel stiffness before and after 50 mM KCl induced VSMC contraction at different pulse pressures.

**Mean pressure (mmHg)**	**Peterson’s modulus (Ep, mmHg)**														
	**20 PP**			**40 PP**			**60 PP**			**80 PP**			**100 PP**		
	**Basal**	**50K**	**ΔEp**	**Basal**	**50K**	**ΔEp**	**Basal**	**50K**	**ΔEp**	**Basal**	**50K**	**ΔEp**	**Basal**	**50K**	**ΔEp**
80	(280 ± 9)	(436 ± 17)****	(156 ± 17)	(259 ± 8)	(362 ± 12)*	(103 ± 13)#	(248 ± 7)	(319 ± 8)	(71 ± 8)	(241 ± 7)	(291 ± 7)	(50 ± 6)	(234 ± 7)	(272 ± 7)	(38 ± 4)
100	(328 ± 11)	(483 ± 17)****	(155 ± 18)	(298 ± 10)	(395 ± 12)*	(96 ± 15)#	(288 ± 12)	(348 ± 9)	(60 ± 10)	(277 ± 11)	(321 ± 9)	(44 ± 8)	(270 ± 10)	(301 ± 9)	(32 ± 6)
120	(437 ± 23)	(560 ± 22)****	(123 ± 22)	(388 ± 22)	(464 ± 16)	(76 ± 19)	(371 ± 22)	(408 ± 14)	(37 ± 13)	(353 ± 20)	(375 ± 14)	(22 ± 10)	(338 ± 18)	(353 ± 13)	(15 ± 7)
140	(625 ± 45)	(695 ± 29)*	(71 ± 29)	(545 ± 42)	(575 ± 27)	(30 ± 22)	(516 ± 41)	(506 ± 25)	(−10 ± 18)	(484 ± 36)	(464 ± 23)	(−20 ± 15)	(454 ± 31)	(434 ± 21)	(−21 ± 11)
160	(894 ± 79)	(863 ± 43)	(−32 ± 41)	(798 ± 71)	(731 ± 43)	(−67 ± 31)	(748 ± 65)	(654 ± 41)*	(−94 ± 26)	(695 ± 60)	(602 ± 38)**	(−93 ± 23)	(645 ± 50)	(562 ± 35)**	(−83 ± 17)
180	(1250 ± 112)	(1079 ± 67)****	(−171 ± 49)	(1158 ± 111)	(938 ± 62)****	(−221 ± 54)	(1089 ± 105)	(854 ± 63)****	(−235 ± 44)	(958 ± 96)	(762 ± 61)****	(−196 ± 38)	(890 ± 84)	(717 ± 57)****	(−174 ± 31)
200	(1705 ± 158)	(1360 ± 98)****	(−345 ± 62)	(1502 ± 140)	(1206 ± 90)****	(−349 ± 63)	(1424 ± 136)	(1056 ± 85)****	(−368 ± 58)	(1339 ± 130)	(990 ± 84)****	(−350 ± 52)	(1277 ± 141)	(880 ± 73)****	(−312 ± 72)

Comparisons are made between the Ep at “50K” and “Basal” (conditions) at the same pulse pressure. Data is expressed as (Mean ± SEM), *n* = 7. *p < 0.05; **p < 0.01; ***p < 0.001; ****p < 0.0001, #*P* < 0.05 (“#” = Compared to the ΔEp at 20PP), Repeated measures two-way ANOVA with Sidak *post hoc* test for multiple comparisons. PP, pulse pressure (mmHg), ΔEp, Ep _50__*K*_–Ep _*Basal*_.

**TABLE 3:** Blood vessel stiffness before and after DEANO-induced VSMC relaxation at different pulse pressures.

**Mean pressure (mmHg)**	**Peterson’s modulus (Ep, mmHg)**														
	**20 PP**			**40 PP**			**60 PP**			**80 PP**			**100 PP**		
	**Basal**	**DEANO**	**ΔEp**	**Basal**	**DEANO**	**ΔEp**	**Basal**	**DEANO**	**ΔEp**	**Basal**	**DEANO**	**ΔEp**	**Basal**	**DEANO**	**ΔEp**
80	(280 ± 9)	(265 ± 6)	(−15 ± 4)	(259 ± 8)	(254 ± 7)	(−5 ± 2)	(248 ± 7)	(248 ± 8)	(0 ± 3)	(241 ± 7)	(242 ± 8)	(1 ± 2)	(234 ± 7)	(238 ± 8)	(4 ± 1)
100	(328 ± 11)	(310 ± 10)	(−18 ± 4)	(298 ± 10)	(297 ± 11)	(−1 ± 3)	(288 ± 12)	(289 ± 13)	(1 ± 3)	(277 ± 11)	(281 ± 12)	(4 ± 3)	(270 ± 10)	(274 ± 11)	(4 ± 2)
120	(437 ± 23)	(409 ± 23)	(−28 ± 4)	(388 ± 22)	(387 ± 23)	(−1 ± 5)	(371 ± 22)	(372 ± 23)	(1 ± 4)	(353 ± 20)	(357 ± 21)	(4 ± 3)	(338 ± 18)	(343 ± 18)	(5 ± 2)
140	(625 ± 45)	(579 ± 43)**	(−46 ± 10)	(545 ± 42)	(543 ± 41)	(−2 ± 7)	(516 ± 41)	(518 ± 41)	(2 ± 6)	(484 ± 36)	(488 ± 37)	(4 ± 6)	(454 ± 31)	(461 ± 32)	(6 ± 4)
160	(894 ± 79)	(844 ± 73)**	(−51 ± 11)	(798 ± 71)	(796 ± 70)	(−2 ± 9)	(748 ± 65)	(752 ± 67)	(3 ± 10)	(695 ± 60)	(700 ± 60)	(5 ± 8)	(645 ± 50)	(653 ± 53)	(8 ± 7)
180	(1250 ± 112)	(1216 ± 113)	(−35 ± 15)	(1158 ± 111)	(1155 ± 110)	(−3 ± 14)	(1089 ± 105)	(1089 ± 102)	(−1 ± 14)	(958 ± 96)	(976 ± 103)	(18 ± 9)	(890 ± 84)	(904 ± 90)	(14 ± 7)
200	(1705 ± 158)	(1659 ± 143)**	(−46 ± 32)	(1502 ± 140)	(1602 ± 146)*	(41 ± 26)	(1424 ± 136)	(1459 ± 151)*	(36 ± 21)	(1339 ± 130)	(1289 ± 147)	(18 ± 16)	(1277 ± 142)	(1211 ± 153)	(19 ± 18)

Comparisons are made between the Ep at “DEANO” and “Basal” (conditions) at the same pulse pressure. Data is expressed as (Mean ± SEM), *n* = 7, *p < 0.05; **p < 0.01. Repeated measures two-way ANOVA with Sidak *post hoc* test for multiple comparisons. PP, pulse pressure (mmHg), ΔEp = Ep_*DEANO*_–Ep _*Basal*_.

Adding DEANO to the aortic segments removed the basal tonus of the VSMCs (Leloup et al., 2018). The comparisons before and after DEANO administration at different pulse pressures are listed in Table 3. Overall, at most pressures, there was no clear effect of removal of basal tonus by DEANO. However, at 20 mmHg pulse pressure Ep was significantly decreased at mean pressures of 140, 160, and 200 mmHg (*p* < 0.01) (Figure 3D). A detailed overview of the effects of 50K and DEANO per pulse pressure can be found in the Supplementary Material (Supplementary Figure 3).

### Comparison of Biomechanical Properties of the Thoracic Descending Aorta and the Abdominal Infrarenal Aorta

**FIGURE 4:**
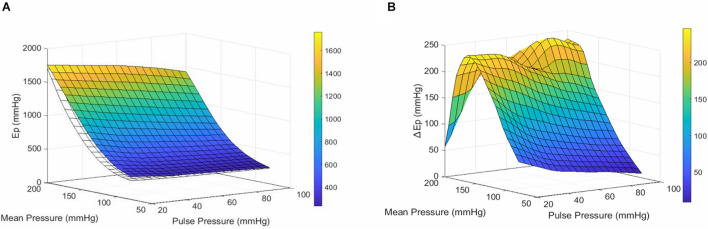
Comparison of the stiffness-profile of the thoracic descending and abdominal infrarenal aorta. **(A)** Surface plot representing the mean Ep of the descending aorta (transparent/white) and the infrarenal aorta (colored) at different pulse & mean pressures. **(B)** Surface plot representing the mean difference in Ep (ΔEP) between the infrarenal and thoracic aorta for every pulse & mean pressure. Ep, Peterson’s modulus of elasticity;ΔEP, EP_infrarenal aorta_–EP_descending aorta_; PP, pulse pressure.

Overall, Ep for the infrarenal artery was higher than Ep for the descending thoracic aorta, indicating a higher stiffness of the infrarenal artery in all conditions. The individual results are listed in Table 4. Whereas differences in Ep between the descending and infrarenal aorta were significant (*p* < 0.05–*p* < 0.01) at mean pressures of 80, 100, 120, and 140 mmHg for a pulse pressure of 20 mmHg, significant differences (*p* < 0.05) were only observed at mean pressures of 100, 120, and 140 mmHg for a pulse pressure of 40 mmHg and at 120 mmHg mean pressure for a pulse pressure of 60 mmHg (Figure 4). At higher pulse pressures, the difference in stiffness between the descending and infrarenal aorta did not reach significance.

**TABLE 4:** Difference in stiffness between the thoracic descending aorta (TDA) and the abdominal infrarenal aorta (AIA) at different pulse and mean pressures.

**Mean pressure (mmHg)**	**Peterson’s modulus (Ep, mmHg)**														
	**20 PP**			**40 PP**			**60 PP**			**80 PP**			**100 PP**		
	**TDA**	**IR**	**ΔEp**	**TDA**	**IR**	**ΔEp**	**TDA**	**IR**	**ΔEp**	**TDA**	**IR**	**ΔEp**	**TDA**	**IR**	**ΔEp**
80	(280 ± 9)	(339 ± 15)*	(59 ± 16)	(259 ± 8)	(296 ± 12)	(37 ± 14)	(248 ± 7)	(274 ± 11)	(26 ± 13)	(241 ± 7)	(259 ± 10)	(18 ± 14)	(234 ± 7)	(242 ± 8)	(9 ± 12)
100	(328 ± 11)	(430 ± 17)**	(102 ± 21)	(298 ± 10)	(368 ± 15)*	(70 ± 21)	(288 ± 12)	(343 ± 13)	(55 ± 20)	(277 ± 11)	(322 ± 12)	(45 ± 19)	(270 ± 10)	(306 ± 11)	(36 ± 17)
120	(437 ± 23)	(599 ± 25)**	(162 ± 35)	(388 ± 22)	(502 ± 18)*	(114 ± 32)	(371 ± 22)	(465 ± 16)*	(94 ± 31)	(353 ± 20)	(431 ± 14)	(78 ± 28)	(338 ± 18)	(406 ± 13)	(69 ± 25)
140	(625 ± 45)	(835 ± 38)*	(211 ± 49)	(545 ± 42)	(727 ± 26)*	(182 ± 48)	(516 ± 41)	(666 ± 22)	(150 ± 47)	(484 ± 36)	(614 ± 20)	(130 ± 43)	(454 ± 31)	(566 ± 16)	(112 ± 37)
160	(894 ± 79)	(1135 ± 55)	(241 ± 76)	(798 ± 71)	(1033 ± 40)	(235 ± 71)	(748 ± 65)	(950 ± 39)	(201 ± 70)	(695 ± 60)	(875 ± 32)	(180 ± 63)	(645 ± 50)	(805 ± 28)	(160 ± 55)
180	(1250 ± 112)	(1452 ± 64)	(202 ± 111)	(1158 ± 111)	(1368 ± 60)	(210 ± 106)	(1089 ± 105)	(1288 ± 56)	(198 ± 105)	(958 ± 96)	(1202 ± 49)	(246 ± 97)	(890 ± 84)	(1114 ± 41)	(226 ± 85)
200	(1705 ± 158)	(1761 ± 84)	(57 ± 160)	(1502 ± 140)	(1695 ± 79)	(182 ± 143)	(1424 ± 136)	(1636 ± 76)	(211 ± 142)	(1339 ± 130)	(1545 ± 69)	(204 ± 137)	(1277 ± 141)	(1426 ± 64)	(233 ± 158)

Data is expressed as (Mean ± SEM), *n* = 7. *p < 0.05; **p < 0.01. Repeated measures two-way ANOVA with Sidak *post hoc* test for multiple comparisons. Comparisons are made between the TDA and the IR subjected to the same PP. PP, pulse pressure (mmHg), TDA, thoracic descending aorta, IR, abdominal infrarenal aorta.

## Discussion

In this study, we demonstrated that increasing *ex vivo* pulsatile load alters the non-linear relationship between pressure and blood vessel stiffness, making the aortic tissue softer. Additionally, the magnitude of the applied pulsatile load altered the response of VSMCs to a contractile stimulus. Finally, we compared the stiffness of the TDA with that of the abdominal infrarenal aorta (AIA). Interestingly, while it is known that the AIA is a stiffer blood vessel compared to the TDA, the difference between the AIA and the TDA becomes less apparent when higher *ex vivo* pulse pressures are applied. These findings suggest that pulsatile load *ex vivo* alters the properties of aortic tissue and therefore impacts the measurement of arterial stiffness. Importantly, it was not the aim of the present study to unravel the altered molecular/biomechanical mechanisms when changing pulse pressure, but rather how manipulating this neglected parameter influences the *ex vivo* measurement of arterial stiffness. Nonetheless, these findings indicate that pulse pressure can be used as an additional variable parameter to better understand arterial stiffening in subsequent studies.

In our setup, whether a segment oscillates at a pulse pressure of 20 or 100 mmHg, both pulses happen at an equal frequency of 10 Hz. Therefore, a segment oscillating at a pulse pressure of 100 mmHg has a higher deformation rate than a segment oscillating at a pulse pressure of 20 mmHg. Our results are in agreement with previous work demonstrating that deformation rate is inversely correlated with the stiffness of (porcine) aortic segments (Delgadillo et al., 2010). Others have reported dissonant results as well. For example, pulse frequency and heart rate have been found to be positively correlated with the dynamic modulus of arteries and PWV, respectively, Bergel (1961), Learoyd and Taylor (1966), Tan et al. (2018), Spronck et al. (2021). However, most of these studies assess the effect of altered pulse frequency on arterial stiffness, which is different from this study since the pulse frequency was kept constant while the ΔP was altered to simulate different pulse pressures. Hydrogels, microtissues and whole tissues such as lung and liver are known to exhibit strain-modulated alterations in viscoelasticity, attributed to, among others, the disruption of crosslinks, protein unfolding and the movement of fluid (Chaudhuri, 2017; Barenholz-Cohen et al., 2020; Walker et al., 2020). Therefore, by applying a larger pulse pressure *ex vivo*, both the larger deformation rate and the higher strain of the aortic segments would lead to alterations in viscoelasticity, expressed as a decreased stiffness. Which viscous or elastic elements are altered in aortic tissue due to increased deformation rate, is largely unclear. In physics, it is known that increasing the strain rate decreases the viscosity of fluids. Since aortic segments are filled with fluid both intracellularly and in the extracellular matrix, follow-up research should assess how altered internal fluid movement/viscosity (due to a change in deformation rate and maximum strain) affects the stiffness of blood vessels, which is similar to the “poro-viscoelastic model” (Caccavo and Lamberti, 2017). Alternatively, while changes in viscoelasticity due to altered pulse pressure are a plausible explanation for the “softening” of the aortic segments, the presence of the Mullins effect cannot be excluded. The Mullins effect (i.e., softening after an initial deformation) has been described by others within cyclic loading of aortic tissue and differs from viscoelasticity (Sokolis, 2007; Horny et al., 2010; Sarangi, 2010; Weisbecker et al., 2013). The Mullins effect goes with residual strain and has been attributed to the interacting properties of elastin and collagen fibers (Sokolis, 2007; Horny et al., 2010; Sarangi, 2010; Weisbecker et al., 2013). To summarize, we have shown that in our setup, increasing pulse pressure softens aortic tissue, by increasing the strain rate. Accordingly, changing the cyclic pulsatile load modulates the (viscoelastic) properties of blood vessels.

Furthermore, our results suggest that contraction-induced stiffness is dependent on the pulsatile load. At lower pulse pressure, the aortic segment produces a larger response to contractile stimuli (50 mM KCl) and therefore generates a higher apparent stiffness. The opposite is true for higher pulse pressures, where a smaller increase in stiffness is observed after introducing a contractile stimulus. Interestingly, this difference is only noticeable at (relatively) low pressures, where the magnitude of the pulse pressure determines the contraction-induced stiffness. However, at pressures above 160 mmHg, there is no difference between a low or high pulse pressure, yet there is an overall difference between the contracted and uncontracted state. This indicates that at higher pressures, the effect of pulse pressure becomes inferior to the effect of pressure itself. One explanation for this phenomenon could be that the efficacy of contraction is dependent on the applied (pulsatile) strain. At low pressures (100 mmHg), the amplitude of the stretch is mainly determined by the pulse pressure. However, at high pressures (200 mmHg), the pressure itself mainly determines the maximum stretch of the aortic segment while the pulse pressure is not able to change the distension much more, causing smaller oscillations (data not shown). This rationale is in line with previously published findings, where the amplitude of the applied stretch controls intracellular calcium levels as well as the reactivity of aortic VSMCs to contractile stimuli (Monaghan et al., 2011; Hutcheson et al., 2012; Leloup et al., 2017). Moreover, mechanical stretch modulates the contractile apparatus of VSMCs, reorganizing the cytoskeleton and promoting focal adhesion (FA) growth (Halka et al., 2008; Chen et al., 2013; De, 2018; Chatterjee et al., 2019). Therefore, rapid responses to altered stretch, such as both FA kinase and ERK1/2 phosphorylation, could explain why adjusting pulse pressure *ex vivo* modulates the excitability of VSMCs toward contraction, but only at low, physiological pressures (Chen et al., 2013; Hu et al., 2014).

Besides the pronounced effect on VSMC contraction-induced stiffness, small, but significant, differences were observed upon removal of basal VSMC tonus by DEANO. Whether these small effects have any physiological relevance is unclear but should not be ruled out. Future research should investigate whether basal tonus is truly affected by pulsatile load.

In the first set of experiments, we focused on the TDA, which is mainly a very elastic artery. The TDA has an important physiological role in dampening the pressure wave generated by the left ventricle. In contrast, the AIA is generally a stiffer blood vessel, having a higher collagen to elastin ratio compared to the TDA (Bia et al., 2005; Cuomo et al., 2017). In the present study, we demonstrated *ex vivo* that the AIA is stiffer than the TDA within the same animal at pressures ranging from 80 to 140 mmHg, but only when low pulse pressures were applied (20–60 mmHg). At higher pulsatile loads, the effect was less pronounced and did not reach statistical significance, in terms of Ep. This means that increased pulsatile load differentially affects the mechanical properties of the AIA and the TDA, and, in essence, shows how *ex vivo* pulsatile load affects the comparison in stiffness between different tissues.

To date, it has not been fully elucidated what biomechanical mechanisms are modulated by *ex vivo* pulse pressure and how they translate to our observed differences and cardiovascular pathologies in general. It is generally accepted that arterial stiffness contributes to an increased pulse pressure *in vivo* (Mitchell et al., 2004, 2010; van der Bruggen M. et al., 2021). In the current work, we have investigated whether there is a reverse crosstalk between pulse pressure and artery stiffness, which is difficult to unequivocally assess *in vivo*. One study (Said et al., 2018) reported that pulse pressure is an independent predictor of CVD mortality. Similarly, a retrospective study based on the data of the Framingham Heart Study has shown that the forward wave amplitude of the blood pressure, but not the mean arterial pressure, is associated with CVD events (Cooper et al., 2015). While these studies suggest an important role of pulsatile load in CVD, the observational design of these studies, make it difficult to determine whether these are causal relationships. On the other hand, observations in orthotopic heart transplant patients provide complementary insights. Patients equipped with a continuous-flow left ventricular assist device (LVAD) show increased aortic stiffness in the first year after transplantation, opposed to patients who received a pulsatile LVAD (Patel et al., 2017). Additionally, acute aerobic exercise, a condition in which pulse pressure is increased due to increased stroke volume, is known to increase arterial compliance (Kingwell et al., 1997; Tanaka et al., 2000; Maeda et al., 2008; Sharman and LaGerche, 2015). In general, these findings provide direct evidence that pulsatile load affects aortic properties, which is in line with our findings. Therefore, future research should investigate how (dynamic) pulsatile load affects mechanical properties of the aortic wall to enhance our understanding of blood vessel micro- and macromechanics (Bia et al., 2005).

## Limitations

While the standard for measuring stiffness of arterial tissue *ex vivo* is evaluation of stress-strain relationships (Butlin et al., 2020), the nature of our ROTSAC set-up does not allow to assess vascular wall stress, since the wall thickness/cross-sectional area cannot be measured. Alternatively, the Peterson’s modulus (Ep) is another, widely used, metric to evaluate blood vessel stiffness (Gosling and Budge, 2003; Santelices et al., 2007; Spronck and Humphrey, 2019). The parameters necessary to calculate Ep are easy to obtain, after translating force into pressure using the Laplace relationship. Ep integrates changes in D0 and ΔD which were both manipulated by the applied pressure protocol. Previous publications from our research group have demonstrated that Ep is a valid metric yielding similar results to other commonly used metrics for arterial stiffness (Leloup et al., 2016; De Munck et al., 2020; Bosman et al., 2021). Another limitation relates to the non-randomized order in which the mean pressures and pulse pressures were changed due to practical reasons. However, pilot experiments did not reveal significant crossover effects. Lastly, all experiments were performed at a frequency of 10 Hz, reflecting the physiological heart rate of mice. From a theoretical perspective, investigating different frequencies would be interesting but beyond the scope of the current work.

## Conclusion

We demonstrated that pulse pressure (i.e., pulsatile load) is a valuable parameter when assessing *ex vivo* blood vessel stiffness. Altering the amplitude of the cyclic oscillations not only modulates the general stiffness of the tissue but also affects the physiological function of VSMCs. While more research is needed to investigate the mechanisms responsible for altered stiffness due to changes in pulsatile load, pulse pressure provides additional insight into the capacity of a blood vessel to resist (cyclic) deformation.

## Data Availability Statement

The original contributions presented in the study are included in the article/Supplementary Material, further inquiries can be directed to the corresponding author/s.

## Author Contributions

CN, WM, and P-JG designed the study. CN and GC performed the experiments. CN drafted the manuscript. PP, GD, WM, and P-JG critically read and revised the manuscript. All authors contributed to the article and approved the submitted version.

## Conflict of Interest

The authors declare that the research was conducted in the absence of any commercial or financial relationships that could be construed as a potential conflict of interest.

## Publisher’s Note

All claims expressed in this article are solely those of the authors and do not necessarily represent those of their affiliated organizations, or those of the publisher, the editors and the reviewers. Any product that may be evaluated in this article, or claim that may be made by its manufacturer, is not guaranteed or endorsed by the publisher.

## Supplementary Material

Supplementary Figure 1 The pulse pressure protocol. The mean pressure was increased from 80 to 200 mmHg in steps of 20 mmHg. This was done five times, with each time a different pulse pressure. The order of pulse pressures (PP) was the same between all experiments and started with a PP of 20 mmHg and ended with a PP of 100 mmHg. The same pulse pressure protocol was applied to each vessel segment in three different conditions (Krebs, KCl, and DEANO). There was a waiting period of 30 min between conditions.

Supplementary Figure 2 Pulse pressure alters diastolic diameter (D0) and ΔP/ΔD Increasing pulse pressure decreases both **(A)** the diastolic diameter (D0) and **(B)** the inverse of the compliance (ΔP/ΔD) which both contribute to the decrease in **(C)** Ep due to increasing pulse pressure. Data is expressed as (Mean ± SEM), *n* = 7, ^∗^*p* < 0.05; ^∗∗^*p* < 0.01; ^∗∗∗^*p* < 0.001, ^****^*p* < 0.0001 Repeated measures One-Way ANOVA with Holm-Sidak *post hoc* test for multiple comparisons [data is compared to; control (= 40 mmHg)]. ΔP/ΔD, inverse of compliance; D0, Diastolic diameter; Ep, Peterson’s modulus of elasticity.

Supplementary Figure 3 The effect of VSMC contraction and basal tonus on Ep per pulse pressure. The relationship between stiffness and pressure (Ep–Mean pressure) under contracting and relaxing conditions at a pulse pressure of **(A)** 20 mmHg, **(B)** 40 mmHg, **(C)** 60 mmHg, **(D)** 80 mmHg, and **(E)** 100 mmHg. Ep, Peterson’s modulus of elasticity; 50K, 50 mM KCl; PP, Pulse pressure.
