# Supplementary figures and images for: High Pulsatile Load Decreases Arterial Stiffness: An ex vivo Study
Source: Front Physiol. 2021 Oct 22;12:741346. doi: 10.3389/fphys.2021.741346 (PMC8569808; doi:10.3389/fphys.2021.741346)

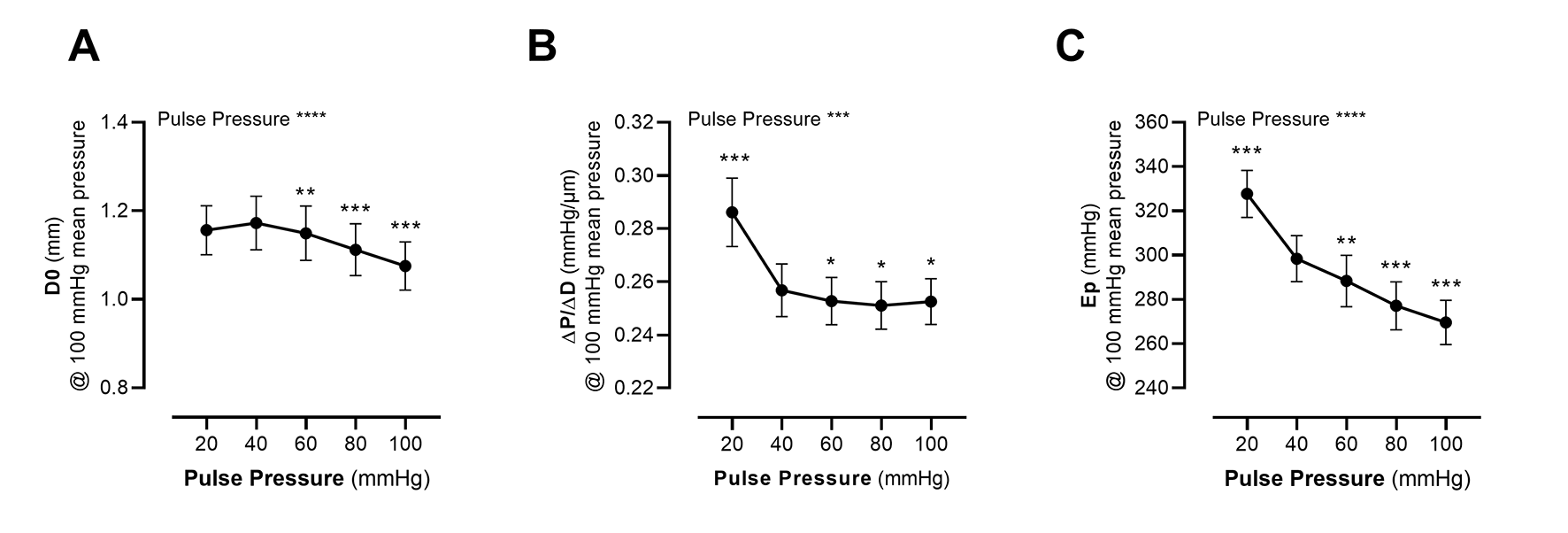

Supplement: Supplementary Figure 2 — Pulse pressure alters diastolic diameter (D0) and ΔP/ΔD Increasing pulse pressure decreases both (A) the diastolic diameter (D0) and (B) the inverse of the compliance (ΔP/ΔD) which both contribute to the decrease in (C) Ep due to increasing pulse pressure. Data is expressed as (Mean ± SEM), n = 7, ∗p < 0.05; ∗∗p < 0.01; ∗∗∗p < 0.001, ****p < 0.0001 Repeated measures One-Way ANOVA with Holm-Sidak post hoc test for multiple comparisons [data is compared to; control (= 40 mmHg)]. ΔP/ΔD, inverse of compliance; D0, Diastolic diameter; Ep, Peterson’s modulus of elasticity. [file Image_2.tif]

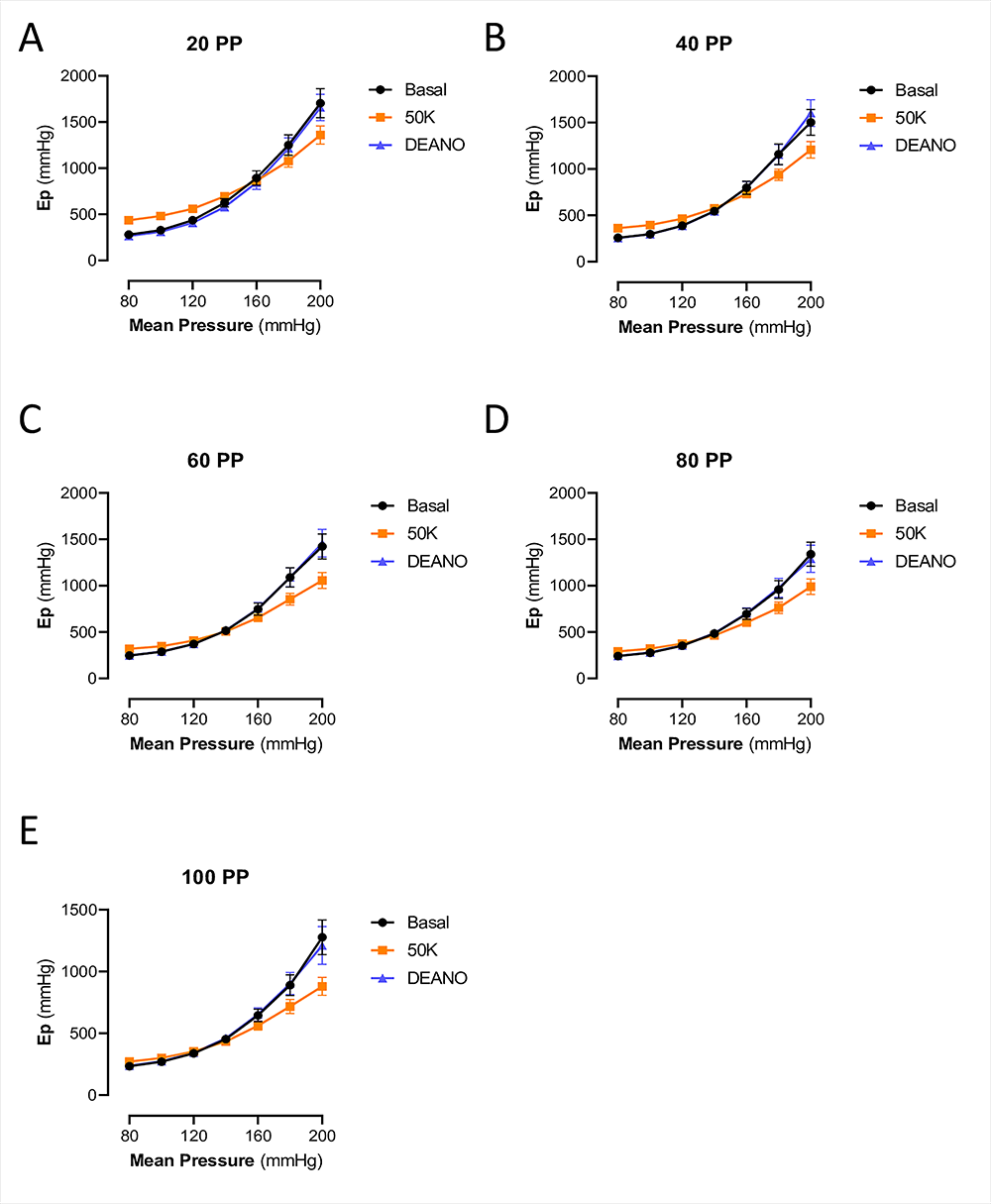

Supplement: Supplementary Figure 3 — The effect of VSMC contraction and basal tonus on Ep per pulse pressure. The relationship between stiffness and pressure (Ep–Mean pressure) under contracting and relaxing conditions at a pulse pressure of (A) 20 mmHg, (B) 40 mmHg, (C) 60 mmHg, (D) 80 mmHg, and (E) 100 mmHg. Ep, Peterson’s modulus of elasticity; 50K, 50 mM KCl; PP, Pulse pressure. [file Image_3.tiff]
